# Association of STarT Back Tool and the short form of the Örebro Musculoskeletal Pain Screening Questionnaire with multidimensional risk factors

**DOI:** 10.1038/s41598-019-57105-3

**Published:** 2020-01-14

**Authors:** Anna Sofia Simula, Olli Ruokolainen, Petteri Oura, Mikko Lausmaa, Riikka Holopainen, Maija Paukkunen, Juha Auvinen, Steven J. Linton, Jonathan C. Hill, Jaro Karppinen

**Affiliations:** 1 Medical Research Center Oulu, Oulu University Hospital and University of Oulu, Oulu, Finland; 2 Faculty of Medicine, Center for Life Course Health Research, University of Oulu, Oulu, Finland; 3 Department of General Medicine Mikkeli Central Hospital (Essote), Mikkeli, Finland; 4 Department of Health Sciences, University of Jyväskylä, Jyväskylä, Finland; 5 Occupational Health Care Center Pihlajalinna, Tampere, Finland; 6 Oulunkaari Health Center, Ii, Finland; 7 Department of Law, Psychology, and Social Work, Center for Health and Medical Psychology, Örebro University, Örebro, Sweden; 8 Institute for Primary Care and Health Sciences, Keele University, Keele, Staffordshire ST5 5BG UK; 9 Finnish Institute of Occupational Health, Oulu, Finland

**Keywords:** Human behaviour, Epidemiology

## Abstract

The Short form of the Örebro Musculoskeletal Pain Screening Questionnaire (ÖMPSQ-short) and the STarT Back Tool (SBT) have been developed to screen for risk factors for future low back pain (LBP) -related disability and work loss respectively. The aim of this study was to investigate the accordance of the two questionnaires and to evaluate the accumulation of risk factors in the risk groups of both screening tools in a large population-based sample. The study population consisted of 3079 participants of the Northern Finland Birth Cohort 1966 who had reported LBP over the previous 12 months and had SBT and ÖMPSQ-short data. We evaluated the association of depressive and anxiety symptoms (Hopkins symptom check list-25, Generalized anxiety disorder 7 questionnaire, and Beck’s Depression Inventory 21), psychological features (Fear-Avoidance Beliefs Questionnaire), lifestyle characteristics (BMI, smoking, alcohol abuse, physical inactivity) and social factors (education level) with the SBT and ÖMPSQ-short risk groups. The high-risk groups of both questionnaires were associated (p < 0.001) with depressive and anxiety symptoms and fear-avoidance beliefs. In addition, adverse lifestyle factors accumulated in the higher risk groups, especially from the ÖMPSQ-short. Agreement between the two questionnaires was moderate for men and fair for women.

## Introduction

Low back pain (LBP) is the most disabling health condition world-wide^1^. No cost-effective or widely available preventive LBP interventions have yet been developed^2^. Predictors of persistent LBP-related disability include symptom-related factors such as previous LBP episodes, pain intensity and the presence of leg pain; lifestyle factors such as overweight/obesity, smoking and physical inactivity; psychological factors such as depression, catastrophizing and fear-avoidance beliefs; and social factors such as education, physical workload and work satisfaction^3^. To improve the effectiveness of healthcare, care processes need to take these factors into account individually and systematically^2^. Early identification of patients who are at the highest risk of developing a prolonged or persistent pain problem is important^2^. The short form of the Örebro Musculoskeletal Pain Screening Questionnaire (ÖMPSQ-short) and the STarT Back Tool (SBT) have been developed for the easy and systematic identification of predictive psychosocial and symptom-related factors^4–6^.

The SBT was developed to identify subgroups of patients with non-specific LBP in order to determine which kind of treatment would benefit each patient. Cut-off scores divide patients into low-, medium- and high-risk groups to enable targeted treatment^5^. Stratified care based on the SBT has shown to improve LBP patients´ outcomes and saving costs compared with current best practice in primary care^7^.

The ÖMPSQ-short was developed from the original 25-item longer version for short and easy clinical utility and has shown to be appropriate for clinical and research purposes^6^. The ÖMSPQ and ÖMPSQ-short focus specifically on the psychosocial risk factors of chronic pain such as depressive symptoms and fear-avoidance beliefs, and thus enable the identification of workers at a higher risk of work disability^4,8,9^. A cut-off score is primarily used to identify patients at high risk^6^ but it can also be used to differentiate between low-risk and medium-risk groups^10,11^.

Both SBT and ÖMPSQ have shown to be valid instruments for identifying people at a higher risk of prolonged disabling pain problems or pain-related adverse effects such as work disability^10^. The suitability of these questionnaires for detecting individuals at a probable risk of prolonged disability from LBP among the population is not clear. A head-to-head comparison of the SBT and ÖMPSQ was evaluated in a cross-sectional study among British primary care LBP patients^10^. In the study, the SBT allocated a smaller proportion of primary care patients with LBP into the high-risk group than the ÖMPSQ; 25% vs. 38%, respectively^10^. To the authors’ knowledge, the proportion of participants allocated to the SBT vs ÖMPSQ-short risk groups has not been compared in a population-based sample. Therefore, our aim was to compare the distribution of participants in each of the risk groups with respect to both of these screening instruments in the Northern Finland Birth Cohort 1966 (NFBC1966), a large birth cohort representing the Northern Finnish general population. Furthermore, we aimed to evaluate the accumulation of psychiatric, psychological, lifestyle and social factors in SBT and ÖMPSQ-short high-risk groups to indirectly validate the questionnaires among the working-age population with LBP.

## Methods

### Study population

The study population belongs to the NFBC1966^12^, which comprises mothers from the two northernmost provinces of Finland, Oulu and Lapland, who had expected dates of delivery in 1966. The original study population included 12 068 mothers and 12 231 children, which was 96.3% of all births in the area during 1966. The population has been followed longitudinally since their antenatal clinic visit, through repeated follow–ups. At the latest data collection point in 2012‒2014, when the cohort members were aged 46, the questionnaires covered lifestyle factors, musculoskeletal symptoms and psychological characteristics. Weight and height were measured in a clinical health examination.

### SBT

The previously validated Finnish version of the SBT was used^13^. The SBT consists of nine independent prognostic indicators of the persistence of disabling LBP, and covers eight constructs: bothersomeness, referred leg pain, comorbid pain, disability (two questions), catastrophizing, fear, and anxiety, and depressive symptoms. The response alternatives to Items 1–8 are “agree = 1 point” or “disagree = 0 point”. Item 9 has five options, of which the two highest responses counted as one point. Thus, the maximum total score range is 0–9. In addition, the psychosocial subscale is derived from Questions 5–9 (range 0–5). The following risk groups were formed: (1) Low-risk (total score of 3 or less); (2) Medium-risk (total score 4 or more and psychosocial subscale score of (3 or less); and (3) High-risk (total score and psychosocial subscale score of 4 or more)^5^ (Supplementary Table 1).

### ÖMSPQ-short

A ten-item short version was formed using the validated Finnish version of the full ÖMPSQ^14^. The questionnaire includes items about (1) the duration of pain(s), (2) pain rating, (3) the ability to do light work, (4) the ability to sleep at night, (5) anxiety feelings, (6) depressed feelings, (7) the perceived risk of pain becoming chronic, (8) self-estimate of return to work and (9‒10) fear-avoidance beliefs. The items are scored from 0 to 10, 0 being the absence of impairment and 10 severe impairment. For Questions 3, 4 and 8, reverse scoring is used. The scores were summed up and the respondents were divided into three groups according to the total score: (1) Low-risk (0‒39 points), (2) Medium-risk (40‒49 points) and (3) High-risk (50‒100 points)^6,10,11^ (Supplementary Table 2).

### Psychiatric and psychological characteristics

We evaluated the presence of depressive and anxiety symptoms in the risk groups using the Hopkins symptom check list-25 (HSCL-25), the Generalized anxiety disorder 7 questionnaire (GAD-7), and Beck’s Depression Inventory 21 (BDI-21). The HSCL-25 is a screening instrument designed to identify common psychiatric symptoms, scored on a scale from 1 (no distress) to 4 (extremely distressed)^15^. The GAD-7 is used as a screening tool and severity measure for generalized anxiety disorder^16^. The BDI-21 is used as a depression inventory^17^. For the HSCL-25, we used a cut-off of <1.55 and ≥1.55 to denote mild and severe mental distress groups, respectively^18^, for the GAD-7 a cut off of <10 and ≥10^16^, and for the BDI-21 a cut off of <14 and ≥14^19^. We also used the Fear-Avoidance Beliefs Questionnaire (FABQ), which contains 16 questions, each scoring from 0 to 6 points. Higher values indicate increased fear-avoidance beliefs^20^. Two subscales exist: A seven-item work subscale (FABQ-W; range, 0–42 points) and a four-item physical activity subscale (FABQ-P; range, 0–24 points)^21^. For the FABQ-W we used a cut-off score of 20 points or less to indicate a low risk, 21 to 24 points to indicate a medium risk, and 25 points or more to indicate a high risk of poor long-term prognosis^22^. For the FABQ-P we used a cut-off of less than 14 points to indicate a low risk, 14 to 15 points to indicate a medium risk, and of 16 points and more to indicate a high risk of poor long-term prognosis^21^. The FABQ has been previously validated in Finnish^23^.

### Lifestyle characteristics

The following lifestyle characteristics were evaluated in the risk groups: body mass index (BMI), smoking, alcohol abuse and physical inactivity. We objectively measured height and weight and calculated BMI (kg/m^2^) using the following category cut-offs: underweight <18.50 kg/m^2^, normal range 18.50‒24.99 kg/m^2^, overweight 25.00‒29.99 kg/m^2^, and obese ≥30.00 kg/m^2^. The underweight and the normal weight group were combined because of the small underweight group size. Participants were categorized into non-smokers, former smokers, and current smokers, using the questions ‘Have you ever smoked regularly?’ and ‘Do you currently smoke?’ Non-smoker and former smoker groups were combined for analyses. We asked the participants how much and how often they consumed beer and other mild alcohol products, wine and spirits. The frequency scale for each alcohol product was: (1) Never, (2) once a year, (3) a few times a year, (4) 3–4 times a year, (5) once every few months, (6) once a month, (7) a few times a month, (8) once a week, (9) a few times a week and (10) daily. The quantity of each alcohol product was measured in units of alcohol: (1) Less than one unit, (2) 1 unit, (3) 2 units, (4) 3 units, (5) 4–5 units, (6) 6–9 units, (7) 10–14 units and (8) 15 units or more. Alcohol units were a bottle for mild alcohol products, a glass for wine and a restaurant unit (about 4cl) for spirits. Total alcohol consumption was calculated in grams of EtOH per day. The cut-off values used to define alcohol abuse were 40 g/d or more for men and 20 g/d or more for women^24^. The level of leisure-time physical activity was elicited by the question: ‘How often do you participate in brisk physical activity/exercise (at least some sweating and breathlessness) during your leisure time?’ which had the following answer options: (1) Once a month or less often, (2) 2‒3 times a month, (3) once a week, (4) 2‒3 times a week, (5) 4‒6 times a week and (6) daily. The participants were classified into two groups depending on their frequency of brisk exercise: Physically active (once a week or more often) and Inactive (less than once a week).

### Social characteristics

We enquired about years of education, including comprehensive school, and classified participants into three classes: (1) less than 9 years, (2) 9‒12 years and (3) over 12 years. Compulsory education in Finland lasts nine years. Participants reported their current employment status at the age of 46 by responding to the question ‘Which of the following describes your current employment status best?’ Response options were: (1) permanent full-time employee, (2) permanent part-time employee, (3) temporary full-time employee, (4) temporary part-time employee, (5) full-time self-employed or entrepreneur, (6) part-time self-employed or entrepreneur, (7) fulltime student, (8) part-time student, (9) unemployed for <6 months, (10) unemployed for 6–12 months, (11) unemployed for >12 months, (12) employed/educated through labour market support, (13) laid off or reduced working hours, (14) maternity/paternity leave or parental leave, (15) retired, (16) caring for my own household, (17) other.’ Three categories were formed: unemployed (not including individuals who were laid off or employed/educated through labour market support), working full-time or part-time (including self-employed individuals and entrepreneurs, not including individuals who were laid off or employed/educated by labour market support) and others.

### Statistical methods

Baseline characteristics were analysed using descriptive statistics. The SBT and ÖMPSQ-short risk group agreement was analysed using Cohen-s Kappa test, where <0.20 was considered poor agreement, 0.21–0.40 fair agreement, 0.41–0.60 moderate agreement, 0.61–0.80 good agreement and values over 0.80 very good agreement^25^. Sankey figures were used to visualize the distribution of participants in each of the risk groups with respect to both of these screening instruments.

Class variables were formed for each psychiatric, psychological, lifestyle and social factors using clinically relevant cut offs. The Chi-square test was used to statistically analyse the association between class variables with the ӦMPSQ-short and SBT risk groups. Strength of association was analysed using odds ratios (ORs) and their 95% confidence intervals (95% CIs). Gender differences were tested using the Mann-Whitney U test. For the ÖMPSQ-short individual questions, the non-parametric Mann-Whitney U test was used because of a skewed distribution of responses. The level of statistical significance was set at p = 0.05. The analyses were stratified by gender and carried out using SPSS version 25.

### Ethical approval

The data were accessed and analysed in an encrypted format with anonymous identification codes. Informed consent was collected from the study population. The study protocol followed the Declaration of Helsinki and was approved by the Ethics Committee of the Northern Ostrobothnia Hospital District.

## Results

In total, 7148 cohort members (69% of those invited) answered the questionnaires. Of these, 1421 (19.9% of the respondents) did not respond to the question on the presence of LBP, whereas 3443 (60.1% of the respondents; 1505 men and 1938 women) reported having LBP over the previous 12 months. Of these, 3079 (1331 men and 1748 women) had SBT and ÖMPSQ-short data. Most typically, pain had lasted 4–5 weeks among men and 6–7 weeks among women. Median pain intensity during the previous week was 3 (interquartile range (IQR) 2–4) among men and 3 (IQR 2–5) among women. Table 1 presents the characteristics of the study population.

**Table 1:** Characteristics of study population.

	men	women	missing men/women n
Gender distribution % (n)	43 (1331)	57 (1748)	
SBT total score median (IQR)	1 (1–2)	1 (1–2)	0/0
ÖMPSQ-short total score median (IQR)	23 (15–33)	26 (17–37)	0/0
HSCL-25 median (IQR)	1.24 (1.12–1.48)	1.32 (1.16–1.56)	135/176
HSCL-25 ≥ 1.55% (n)	19.6 (235)	26.1 (410)	
GAD-7 + median (IQR)	1 (0–4)	2 (0–5)	150/142
GAD-7 ≥ 10% (n)	2.0 (24)	3.7 (60)	
BDI-21 median (IQR)	4 (1–8)	5 (2–10)	61/72
BDI-21 ≥ 14% (n)	8.9 (113)	13.5 (227)	
FABQ-P median (IQR)	10 (5–14)	9 (5–14)	19/14
Low risk % (n)	73.1 (959)	74.9 (1298)	
Medium risk % (n)	8.8 (116)	8.7 (150)	
High risk % (n)	18.1 (237)	16.5 (286)	
FABQ-W median (IQR)	9 (2–18)	7 (0–16)	31/54
Low risk % (n)	79.1 (1028)	81.7 (1384)	
Medium risk % (n)	9.3 (121)	8.1 (138)	
High risk % (n)	11.6 (151)	10.2 (172)	
BMI mean (SD)	27.5 (4.3)	26.8 (5.5)	2/3
<25% (n)	27.8 (369)	44.1 (770)	
25–29.99% (n)	49.1 (653)	32.9 (574)	
≥30% (n)	23.1 (307)	23.0 (401)	
Smoking % (n)			93/85
non-smokers	46 (571)	56 (937)	
former smokers	34 (424)	26 (435)	
current smokers	20 (243)	18 (291)	
Alcohol abuse % (n)	8.6 (108)	8.5(143)	77/66
Physical activity % (n)			100/81
Inactive (less than once a week)	33 (402)	25 (421)	
Active (once a week or more often)	62 (829)	71 (1246)	
Education % (n)			134/130
under 9 years	3 (38)	3 (48)	
9–12 years	73 (868)	67 (1078)	
over 12 years	24 (291)	30 (492)	
Working status % (n)			290/298
unemployed	5 (54)	4 (56)	
working full-time or part-time	90 (940)	89 (1283)	
others	5 (47)	8 (111)	

ÖMPSQ-short (Örebro Musculoskeletal Pain Screening Questionnaire short form), SBT (Start Back Tool), IQR (interquartile range), HSCL-25 (Hopkins symptom check list-25), GAD-7 (Generalized anxiety disorder 7 questionnaire), BDI-21 (Beck’s Depression Inventory 21), FABQ-P (Fear avoidance beliefs questionnaire physical activity subscale), FABQ-W (Fear-avoidance beliefs questionnaire work subscale), BMI (body mass index).

**Figure 1:**
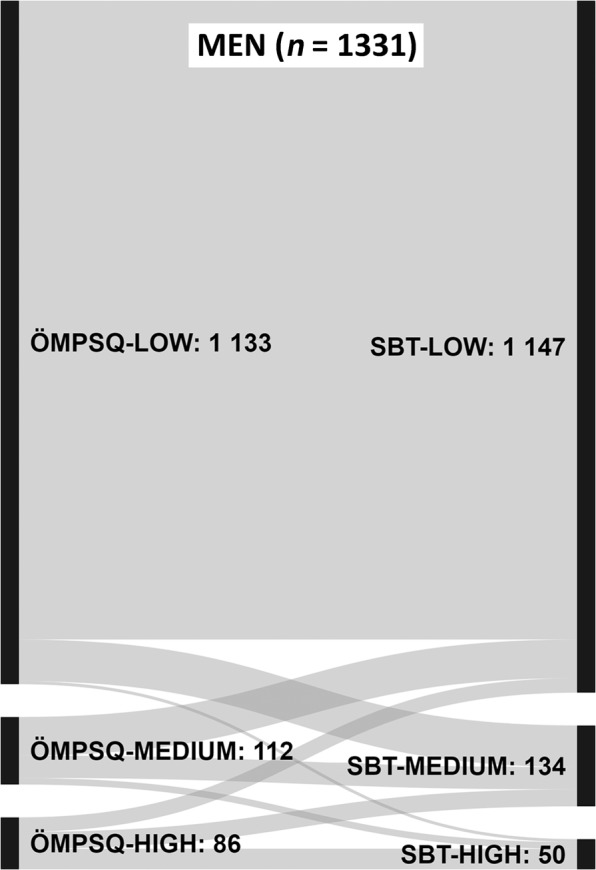
Sankey plots representing the accordance of the SBT (Start Back Tool) and ÖMPSQ-short (Örebro Musculoskeletal Pain Screening Questionnaire) risk groups among men.

**Figure 2:**
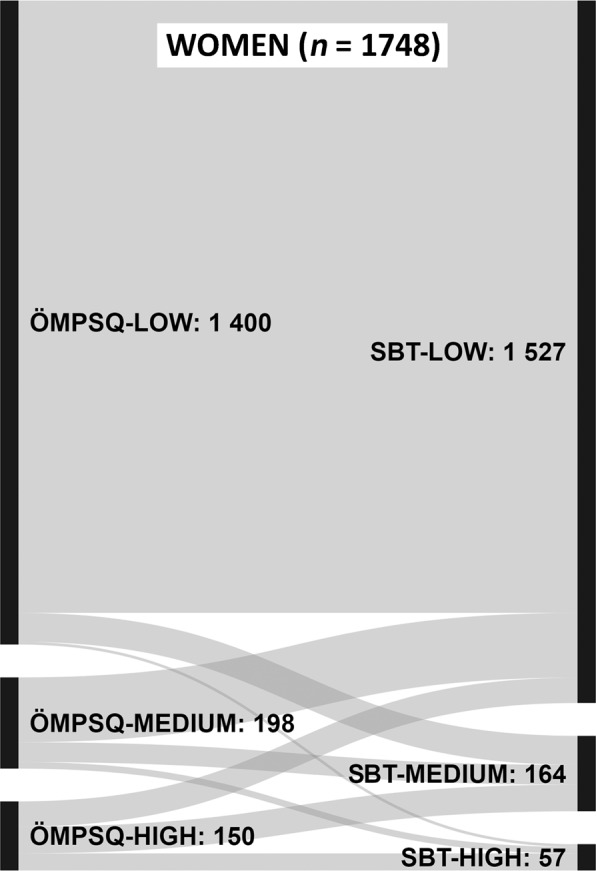
Sankey plots representing the accordance of the SBT (Start Back Tool) and ÖMPSQ-short (Örebro Musculoskeletal Pain Screening Questionnaire) risk groups among women.

Tables 2 and 3 present individual question scores and the distribution of participants in the ÖMPSQ-short and SBT risk groups in the study population (histograms in Supplementary Data Figs. 1–10). Significant gender difference in risk group allocation was seen using ÖMPSQ (p = 0.001), but not using SBT (p = 0.600). According to ÖMPSQ-short 85% of men and 80% of women were allocated to the low-risk group compared to 86% and 87%, respectively, according to SBT. ÖMPSQ-short allocated 8% of men and 11% of women to medium-risk group, while the corresponding percentages using SBT were 10% and 9%, respectively. Women were significantly more often represented in the ÖMPSQ-short high-risk group (men 7%, women 9%; p = 0.028) but not in the SBT high-risk group (men 4%, women 3%; p = 0.457). The agreement of the SBT and ÖMPSQ-short risk groups are presented in Figs. 1 and 2. The agreement of the SBT and ÖMPSQ-short risk groups was moderate for men (Kappa value 0.409; 95% confidence interval (CI) 0.348–0.470, p < 0.001) and fair for women (Kappa value 0.328; 95% CI 0.281–0.375, p < 0.001).

**Table 2:** Single question medians of ÖMPSQ-short questions and distribution of study participants in the risk groups.

ÖMPSQ-short	Men (n = 1331) median (IQR)	Women (n = 1748) median (IQR)	p-value for gender difference
1. How long have you had your current pain problem? Tick (√) one.	3 (1–10)	4 (1–10)	0.190
2. How would you rate the pain that you have had during the past week? Circle one.	3 (2–4)	3 (2–5)	**<0.001**
3. I can do light work (or home duties) for an hour.	0 (0–1)	0 (0–1)	0.545
4. I can sleep at night.	0 (0–2)	1 (0–3)	**<0.001**
5. How tense or anxious have you felt in the past week? Circle one.	2 (1–4)	3 (1–5)	**<0.001**
6. How much have you been bothered by feeling depressed in the past week? Circle one.	0 (0–2)	1 (0–2)	**<0.001**
7. In your view, how large is the risk that your current pain may become persistent?	3 (1–6)	4 (1–7)	**0.002**
8. In your estimation, what are the chances you will be working your normal duties (at home or work) in 3 months?	0 (0–1)	0 (0–1)	0.090
9. An increase in pain is an indication that I should stop what I’m doing until the pain decreases.	6 (2–9)	6 (2–8)	0.346
10. I should not do my normal work (at work or home duties) with my present pain.	0 (0–2)	0 (0–2)	0.853
Risk group % (n)			**0.001**
low	85.1 (1133)	80.1 (1400)	
medium	8.4 (112)	11.3 (198)	
high	6.5 (86)	8.6 (150)	**0.028**

For all 10 questions, a higher number indicates a greater impairment. Gender differences were tested using the Mann-Whitney U test for the ÖMPSQ-short individual questions and the Chi-square test for the risk groups. ÖMPSQ (Örebro Musculoskeletal Pain Screening Questionnaire).

**Table 3:** Percentages of ‘agree’ responses to SBT Questions 1–8 and percentages of two highest response options for Question 9, and the distribution of study participants in the risk groups.

SBT	Men (n = 1331) % (n)	Women (n = 1748) % (n)	p-value for gender difference
1. Has your back pain spread down your leg(s) at some time in the last 2 weeks?	29 (382)	32 (560)	**0.047**
2. Have you had pain in the shoulder or neck at some time in the last 2 weeks?	61 (810)	74 (1291)	**<0.001**
3. Have you only walked short distances because of your back pain?	5 (62)	6 (109)	0.058
4. In the last 2 weeks, have you dressed more slowly than usual because of back pain?	11 (149)	10 (166)	0.124
5. Do you think it’s not really safe for a person with a condition like yours to be physically active?	5 (70)	5 (80)	0.383
6. Have worrying thoughts been going through your mind a lot of the time?	19 (255)	21 (373)	0.137
7. Do you feel that your back pain is terrible and it’s never going to get any better?	10 (135)	7 (116)	**<0.001**
8. In general have you stopped enjoying all the things you usually enjoy?	21 (285)	20 (347)	0.288
9. Overall, how bothersome has your back pain been in the last 2 weeks?	7 (92)	8 (132)	0.499
Risk group % (n)			0.600
low	86.2 (1147)	87.4 (1527)	
medium	10.1 (134)	9.4 (164)	
high	3.8 (50)	3.3 (57)	0.457

Gender differences were tested using the Chi-square test. SBT (Start Back Tool, IQR (interquartile range), Q1–10 (Question 1–10).

### Association between risk groups and psychiatric characteristics

Table 4 shows the associations between the SBT and ÖMPSQ-short risk groups and psychiatric and psychological characteristics. Clinically relevant depressive and anxiety symptoms and fear-avoidance beliefs increased from the low- to medium-risk groups and from the medium- to high-risk groups using both the SBT and ÖMPSQ-short, among both men and women (p < 0.001) and by all indicators (HCSL-25, GAD-7, BDI-21, FABQ-P and FABQ-W).

**Table 4:** Association between SBT or ÖMPSQ-short risk groups and psychiatric and psychological characteristics, analysed using the Chi-square test.

	SBT % (n)				ÖMPSQ-short % (n)			
	Low-risk	Medium-risk	High-risk	P value	Low-risk	Medium- risk	High-risk	P value
**Men**								
HSCL-25 ≥ 1.55	16 (167)	40.7 (48)	51.3 (20)	<0.001	15.2 (156)	36.4 (36)	58.1 (43)	<0.001
GAD-7 ≥ 10	1.1 (11)	5.9 (7)	14.3 (6)	<0.001	0.9 (9)	3.2 (3)	15.6 (12)	<0.001
BDI-21 ≥ 14	6.2 (68)	22.2 (28)	36.2 (17)	<0.001	5.2 (57)	19.2 (20)	45.0 (36)	<0.001
FABQ-P				<0.001				<0.001
Low risk	77.1 (871)	55.6 (74)	28 (14)		78.1 (871)	51.8 (58)	35.3 (30)	
Medium risk	8.3 (94)	14.3 (19)	6.0 (3)		8.2 (91)	14.3 (16)	10.6 (9)	
High risk	14.5 (164)	30.1 (40)	66.0 (33)		13.7 (153)	33.9 (38)	54.1 (46)	
FABQ-W				<0.001				<0.001
Low risk	83.3 (935)	56.6 (73)	41.7 (20)		85.0 (943)	54.1 (59)	32.1 (26)	
Medium risk	7.8 (88)	22.5 (29)	8.3 (4)		7.1 (79)	23.9 (26)	19.8 (16)	
High risk	8.9 (100)	20.9 (27)	50.0 (24)		7.9 (88)	22.0 (24)	48.1 (39)	
**Women**								
HSCL-25 ≥ 1.55	23.3 (322)	40.7 (60)	51.3 (28)	<0.001	19.7 (249)	43.3 (77)	66.1 (84)	<0.001
GAD-7 ≥ 10	2.5 (35)	11.4 (17)	15.1 (8)	<0.001	1.6 (21)	8.4 (15)	18.3 (24)	<0.001
BDI-21 ≥ 14	9.9 (145)	35.5 (55)	50.9 (27)	<0.001	7.9 (107)	22.5 (41)	54.5 (79)	<0.001
FABQ-P				<0.001				<0.001
Low risk	78.8 (1194)	53.1 (86)	32.1 (18)		79.9 (1110)	59.6 (118)	47.6 (70)	
Medium risk	7.9 (120)	13.6 (22)	14.3 (8)		7.7 (107)	13.1 (26)	11.6 (17)	
High risk	13.3 (202)	33.3 (54)	53.6 (33)		13.7 (172)	27.3 (54)	40.8 (60)	
FABQ-W				<0.001				<0.001
Low risk	84.8 (1258)	67.1 (104)	39.3 (22)		87.5 (1190)	65.8 (127)	47.5 (67)	
Medium risk	7.4 (110)	13.5 (21)	12.5 (7)		6.7 (91)	15.0 (29)	12.8 (18)	
High risk	7.8 (115)	19.3 (30)	48.2 (27)		5.8 (79)	19.2 (37)	39.7 (56)	

ÖMPSQ (Örebro Musculoskeletal Pain Screening Questionnaire), SBT (Start Back Tool), HSCL-25 (Hopkins symptom check list-25), GAD-7 (Generalized anxiety disorder 7 questionnaire), BDI-21 (Beck’s Depression Inventory), FABQ-P (Fear-avoidance beliefs questionnaire physical activity subscale), FABQ-W (Fear-avoidance beliefs questionnaire work subscale).

#### Lifestyle and social characteristics

Table 5 shows the association between the risk groups and lifestyle and social characteristics. The men and women in the SBT high-risk group were more likely to be obese (odds ratio (OR) 2.3 (95% CI 1.3–4.1) for men and 2.2 (1.3–3.7) for women) and current smokers (OR 3.0 (1.6–5.8) for men and 1.8 (0.99–3.3) for women) than those in the low- and medium-risk groups. In addition, the men in the SBT high-risk group were more likely to consume alcohol over the risk limits (OR 2.5; 1.1–5.5) and less likely to have been in education for over 12 years (OR 0.4; 0.17–1.1) than those in the low- and medium-risk groups. Among the women in the SBT high-risk group, the proportion of participants who had been in education for 12 years or more was the smallest (OR 0.2; 0.2–0.8). Physically inactive women were more likely to be allocated to the SBT high-risk group (OR 2.5; 1.4–5). Physical inactivity among men and alcohol abuse among women did not significantly differ between the SBT risk groups.

**Table 5:** Association between SBT or ÖMPSQ-short risk groups and lifestyle and social characteristics, analysed using the Chi-square test.

	SBT % (n)			P value	ÖMPSQ-short % (n)			P value
	Low-risk	Medium-risk	High-risk		Low-risk	Medium-risk	High-risk	
**Men**								
BMI				**0.014**				**0.016**
<25	28.3 (324)	24.8 (33)	24.0 (12)		28.3 (320)	23.2 (26)	26.7 (23)	
25–29.99	50.1 (574)	45.9 (61)	36.0 (18)		50.2 (568)	42.0 (47)	44.2 (38)	
≥30	21.6 (248)	29.3 (39)	40.0 (20)		21.5 (243)	34.8 (39)	29.1 (25)	
Smoking	16.8 (181)	37.2 (45)	41.5 (17)	**<0.001**	17.0 (179)	28.0 (30)	45.3 (34)	**<0.001**
Alcohol abuse	7.9 (86)	11.4 (14)	18.2 (8)	**0.030**	7.6 (81)	12.1 (13)	17.7 (14)	**0.003**
Physically inactive	31.4 (336)	39.8 (47)	43.1 (19)	0.058	31.3 (328)	32.1 (34)	52.6 (40)	**0.001**
Education years				**<0.001**				**0.002**
<9	2.5 (26)	9.0 (10)	5.0 (2)		2.7 (28)	4.0 (4)	8.0 (6)	
9–12	71.7 (747)	76.5 (88)	82.5 (33)		71.2 (728)	80.0 (80)	80.0 (60)	
>12	25.8 (269)	14.8 (17)	12.5 (5)		26.0 (266)	16.0 (16)	12.0 (9)	
**Women**								
BMI				**<0.001**				**<0.001**
<25	45.9 (699)	32.9 (54)	29.8 (17)		46.6 (651)	36.9 (55)	32.3 (64)	
25–29.99	32.9 (502)	32.9 (54)	31.6 (18)		32.3 (452)	29.5 (44)	39.4 (78)	
≥30	21.3 (323)	34.1 (56)	38.6 (22)		21.2 (295)	33.6 (50)	28.3 (56)	
Smoking	16.2 (236)	26.5 (40)	27.3 (15)	**0.001**	15.5 (207)	27.9 (39)	24.2 (45)	**<0.001**
Alcohol abuse	8.0 (118)	12.3 (19)	10.9 (6)	0.150	7.9 (106)	17.6 (25)	6.3 (12)	**<0.001**
Physically Inactive	23.4 (342)	35.9 (55)	43.6 (24)	**<0.001**	22.9 (307)	45 (63)	27.0 (51)	**<0.001**
Education years				**<0.001**				**<0.001**
<9	2.3 (33)	6.8 (10)	9.3 (5)		2.3 (30)	8.2 (11)	3.8 (7)	
9–12	66.0 (936)	71.9 (105)	68.5 (37)		65.0 (844)	70.1 (94)	75.7 (140)	
>12	31.7 (449)	21.2 (31)	22.2 (12)		32.7 (425)	21.6 (29)	20.5 (38)	

ÖMPSQ (Örebro Musculoskeletal Pain Screening Questionnaire), SBT (Start Back Screening Tool), BMI (body mass index).

Men in the ÖMPSQ-short high-risk group were more likely to be current smokers (OR 3.8; 2.3–6.1) and to consume alcohol over the risk limits (OR 2.5; 1.3–4.6) and less likely to be physically active (OR 0.4; 0.26–0.66) and less educated (OR 0.4; 0.20–0.83) than those in the low- and medium-risk groups. The women in the ÖMPSQ-short low-risk group were less likely to be obese (OR 0.6; 0.5–0.8), current smokers (OR 0.5; 0.4–0.7), physically inactive (OR 0.5; 0.4–0.7) and most likely to have education of over 12 years (OR 2.0; 1.4–2.5) than those in the medium- and high-risk groups. Among the women, alcohol abuse was most pronounced in the medium-risk group (17.6%) according to the ÖMPSQ-short, and the difference between the low- (7.9%) and high-risk (6.3%) groups was small.

## Discussion

This study aimed to assess the use of the SBT and ÖMPSQ-short tools in a population-based sample of 3079 Northern Finns with LBP. We explored the agreement of the two widely used tools for identifying individuals at a higher risk of prolonged LBP-related disability (SBT) or work disability related to musculoskeletal pain (ÖMPSQ-short) in a large birth cohort. In addition, we assessed the accumulation of known risk factors in risk groups of the questionnaires. In our study, the SBT and ÖMPSQ-short demonstrated fair to moderate agreement with each other and both tools demonstrated varying strengths in their associations with several psychiatric, psychological, lifestyle and social characteristics.

The percentages of the individuals belonging to the medium- and high-risk groups were smaller in the current large cohort than in previous studies of comparatively small LBP patient samples^10,26–28^. Our population-based sample may explain the differences in risk group proportions, compared to patient populations in other studies’ samples. Agreement between the SBT and ÖMPSQ-short risk groups have not been studied earlier in a population-based sample. In terms of individuals reporting LBP during the previous 12 months, an agreement was observed between the SBT and ÖMPSQ-short risk groups, but the questionnaires classified partly different individuals into the risk groups. Among Swedish acute or subacute LBP patients, and among Brazilian primary care LBP patients, a moderate agreement has been observed between the SBT and ÖMPSQ-short risk groups^27,28^. Similar findings were observed in the English study comparing the SBT and ÖMPSQ (24 item)^10^. The ÖMPSQ-short seemed to allocate more people into the high-risk group than the SBT in our study, in accordance with an earlier study of LBP patients^10^. A Swedish study of LBP patients obtained opposite results^28^. However, in the Swedish study the medium- and high-risk groups of SBT were merged, which may explain the contrasting results. Gender differences between the questionnaires has been shown earlier in the Swedish study among patients with LBP as the agreement between the SBT and ÖMPSQ-short was lower among women aged 50 or over than among men of the same age^28^. We discovered gender differences between the risk groups using ÖMPSQ-short but not using SBT. A few responses to single ÖMPSQ-short and SBT questions were different between females and males.

The high-risk groups in both questionnaires had a higher likelihood of having psychiatric symptoms and fear-avoidance beliefs. A cross-sectional study of non-specific LBP patients visiting a GP practice, using the Tampa Scale of Kinesiophobia questionnaire^10^ has also shown a correlation between fear of movement and SBT or ÖMPSQ. Similar results with respect to fear of movement have been observed in later cross-cultural validation studies of SBT^29–32^. SBT total and/or psychosocial subscale scores have shown to be associated with LBP-related disability, bothersomeness, catastrophizing, and depression/ depressive symptoms^5,13,29–35^. Emotional and behavioural problems were strongly associated with multisite musculoskeletal pains in an earlier population-based study^36^. Associations between the SBT and ÖMPSQ-short risk groups and psychiatric and psychological characteristics, using the HSCL-25, GAD-7, BDI-21, FABQ-P and FABQ-W, have not been studied previously in a population-based study. In our study, we applied widely used clinical cut-offs for the analyses. We found a clear association between all the tested psychiatric and psychological outcomes and the SBT or ÖMPSQ-short subgroups among both men and women. This finding is in concordance with the target of both questionnaires to identify individuals with psychosocial risk factors for prolonged disability. Interestingly, the ÖMPSQ-short classified more participants with clinically relevant psychiatric symptoms and fear-avoidance beliefs into the high-risk group than the SBT. Fear-avoidance beliefs are understood as a potentially treatment-modifiable risk factor. Sensitivity to identifying fear avoidance beliefs was higher when the ÖMPSQ was used, but specificity was much lower than when the SBT was used. In the SBT high-risk group, the proportion of individuals with fear avoidance beliefs was greater and the proportion of individuals at a low risk of fear avoidance beliefs was lower than in the ÖMPSQ high-risk group. This may explain why the SBT is an effective and cost-effective screening instrument for targeted care. It would be interesting to use both screening instruments (SBT and ÖMPSQ-short) as part of a prevention strategy for patients with a new episode of LBP seeking healthcare in either an occupational or primary care setting and to evaluate the cost-effectiveness of such a combined screening strategy.

An association between the ÖMPSQ-short risk groups and lifestyle factors (smoking and obesity) and the number of pain sites has also been shown earlier in the same population among individuals reporting musculoskeletal pain during the last 12 months^11^. No other studies have reported an association between lifestyle or social factors and the SBT or ÖMPSQ-short. According to our study of individuals reporting LBP during the last 12 months, the individuals belonging to the medium- and high-risk group of the SBT or ÖMPSQ-short had significantly more adverse health behaviours than the low-risk group members. The lifestyle profile differed between the SBT and ÖMPSQ-short high-risk groups and between genders. BMI was associated with the risk groups of both questionnaires, among both men and women. The likelihood of smoking increased from the low- to the medium-risk group and from the medium- to the high-risk group among men, but the difference between the medium- and high-risk groups was not as clear among women. Physical inactivity was least prevalent in the low-risk group among women using both questionnaires. Among men, physical inactivity was most prevalent in the high-risk group using only the ÖMPSQ-short. In an earlier study of individuals reporting musculoskeletal pain during the last 12 months, the association between physical activity and the ÖMPSQ-short risk classification was not significant^11^. These findings emphasize that inactivity may play a greater role as a risk factor for prolonged disability, and that high activity levels might not automatically play a protective role. Medium activity level was associated with lower prevalence of LBP according a systematic review, while the association between high activity level and LBP, compared to low activity level, was not clear^37^. In a large prospective study consisting of older adults, high-risk individuals, as classified according to 11 biopsychosocial risk factors, were three times more likely to develop pain compared to low-risk group^38^. Interestingly, 7.8% of the study sample belonged to the high-risk group, which is similar to ÖMPSQ-short high-risk group percentages in our study (7% in men, 9% in women). Similar to our study, they also found that individuals in the high-risk group were the least likely to have higher level education. In summary, our results support the significance of lifestyle and social factors among individuals belonging to ÖMPSQ-short and SBT high-risk groups.

A meta-analysis has shown that the ÖMPSQ excellently predicts work absenteeism while SBT does not^39^. This is a clear difference between these questionnaires according to earlier studies. In this study, we found some differences, as the proportion of individuals with psychiatric symptoms was higher in the high-risk group when using the ÖMPSQ-short compared to the SBT. On the other hand, the proportion of individuals at a high risk of fear-avoidance beliefs was larger in the high-risk group when the SBT used. In addition to that, the accumulation of lifestyle and social characteristics in the ÖMPSQ-short high-risk group was more pronounced compared to SBT high-risk group. It would be interesting to evaluate what is the role of lifestyle and social factors in comparison to psychological factors in work absenteeism due to LBP. In health care, the evaluation of lifestyle and social factors, together with psychological characteristics, should be a coherent part of LBP patient assessment in order to identify adverse lifestyle and the behavioural determinants of pain. This may lead to a better LBP outcome and better comprehensive welfare. Indeed, lifestyle intervention, compared to usual care, was shown as cost-effective in a randomized controlled study^40^.

A major strength of our study was the use of a birth cohort, which provided a large population-based sample of 3079 individuals originating from the same geographical area. Moreover, we had a moderately high response rate. The cohort effectively represents the Northern Finnish population as it originally included 96% of the Northern Finnish population born in 1966. The Data on many psychiatric, psychological, lifestyle and social characteristics enabled a comprehensive evaluation of the SBT and ÖMPSQ-short. For data collection, we used validated questionnaires and clinical cut-offs. As the study design was cross-sectional, we could only investigate associations, and not causality. Thus, the predictive potential of the risk allocations of the SBT and the ÖMPSQ-short remain to be evaluated in this population. The cut-off scores for the low- and medium-risk groups of ÖMPSQ have previously been used only according to the 25-item ÖMPSQ, which is considered as a limitation. A second limitation is that we have validated in Finnish only the long form of ÖMPSQ^14^ but, on the other hand, the 10 items of the ÖMPSQ-short form a part of the long form.

## Conclusions

The SBT and ÖMPSQ-short high-risk groups, in a population sample reporting LBP during the last 12 months, manifested clinically relevant depressive and anxiety symptoms and fear-avoidance beliefs, which are known risk factors for prolonged disability and poor LBP outcome. In addition, several adverse lifestyle factors accumulated in the higher risk groups, especially when using the ÖMPSQ-short. We found differences between the two questionnaires mostly related to lifestyle and social factors. SBT is shorter and faster to use and therefore more applicable for example during appointments with time constraints (such as physicians typically have). ÖMPSQ-short is more multifaceted and may better identify abnormal lifestyle factors. How significant the differences between the two questionnaires are requires further investigation. The SBT and ÖMPSQ-short are suitable tools for detecting individuals with accumulated psychiatric, psychological and lifestyle risk factors for prolonged disability due to LBP among working-age people with LBP. Our results justify exploring whether a similar accumulation of risk factors can be detected using the SBT and ÖMPSQ-short in other population-based samples. In cases of very common, disabling and expensive health problems, the prevention of poor outcomes is the most desirable way to reduce the problem and its consequences. In our study, both questionnaires were able to detect several risk factors of poor LBP outcome and therefore a combination of them could be a good option. Further research is needed to assess the effectiveness and cost-effectiveness of targeted preventive interventions using the SBT and/or ÖMPSQ-short.

## Supplementary information

Supplementary information.
