# Supplementary material for: Association of STarT Back Tool and the short form of the Örebro Musculoskeletal Pain Screening Questionnaire with multidimensional risk factors
Source: Sci Rep. 2020 Jan 14;10:290. doi: 10.1038/s41598-019-57105-3 (PMC6959304; doi:10.1038/s41598-019-57105-3)

Supplementary info for the article: Association of STarT Back Tool and the short form of the Örebro Musculoskeletal Pain Screening Questionnaire with multidimensional risk factors

Anna-Sofia Simula, Olli Ruokolainen, Petteri Oura, Bachelor of Medicine, Mikko Lausmaa, Riikka Holopainen, Maija Paukkunen, Juha Auvinen, Steven J. Linton, Jonathan C Hill, Jaro Karppinen.

Supplementary Table 1. *SBT*

<table>
<tr><td colspan="3">Thinking about the last 2 weeks tick your response to the following questions:</td><td>disagree</td><td>agree</td></tr>
<tr><td colspan="3">1. Has your back pain spread down your leg(s) at some time in the last 2 weeks?</td><td>0</td><td>1</td></tr>
<tr><td colspan="3">2. Have you had pain in the shoulder or neck at some time in the last 2 weeks?</td><td>0</td><td>1</td></tr>
<tr><td colspan="3">3. Have you only walked short distances because of your back pain?</td><td>0</td><td>1</td></tr>
<tr><td colspan="3">4. In the last 2 weeks, have you dressed more slowly than usual because of back pain?</td><td>0</td><td>1</td></tr>
<tr><td colspan="3">5. Do you think it’s not really safe for a person with a condition like yours to be physically active?
6. Have worrying thoughts been going through your mind a lot of the time?</td><td>0</td><td>1</td></tr>
<tr><td colspan="3">7. Do you feel that your back pain is terrible and it’s never going to get any better?</td><td>0</td><td>1</td></tr>
<tr><td colspan="3">8. In general have you stopped enjoying all the things you usually enjoy?</td><td>0</td><td>1</td></tr>
<tr><td colspan="5"></td></tr>
<tr><td colspan="5">9. Overall, how bothersome has your back pain been in the last 2 weeks?</td></tr>
<tr><td>Not at all 0</td><td>Slightly 0</td><td>Moderately 0</td><td>Very much 1</td><td>Extremely 1</td></tr>
<tr><td colspan="5"></td></tr>
<tr><td colspan="5">For questions 1–8, disagree is calculated as 0 point and agree is calculated as 1 point. For Question 9, the responses very much or extremely are calculated as 1 point. The total score is the sum of all nine questions, the sub score sum of Questions 5–9.</td></tr>
</table>

Supplementary Table 2. ÖMPSQ-short

1. How long have you had your current pain problem? Tick (√) one.<br><br>*0–1 weeks [1] 1–2 weeks [2] 3–4 weeks [3] 4–5 weeks [4] 6–8 weeks [5]*<br>*9–11 weeks [6] 3–6 months [7] 6–9 months [8] 9–12 months [9] over 1 year [10]*
2. How would you rate the pain that you have had during the past week? Circle one.<br><br>*0 1 2 3 4 5 6 7 8 9 10*<br>*No pain Pain as bad as it could be*
For Items 3 and 4, please circle the one number that best describes your current ability to participate in each of these activities.
3. I can do light work (or home duties) for an hour.<br><br>*0 1 2 3 4 5 6 7 8 9 10 (10-)*<br>*Not at all Without any difficulty*
4. I can sleep at night.<br>*0 1 2 3 4 5 6 7 8 9 10 (10-)*<br>*Not at all Without any difficulty*
5. How tense or anxious have you felt in the past week? Circle one.<br>*0 1 2 3 4 5 6 7 8 9 10*<br>*Absolutely calm and relaxed As tense and anxious as I've ever felt*
6. How much have you been bothered by feeling depressed in the past week? Circle one.<br>*0 1 2 3 4 5 6 7 8 9 10*<br>*Not at all Extremely*
7. In your view, how large is the risk that your current pain may become persistent?<br>*0 1 2 3 4 5 6 7 8 9 10*<br>*No risk Very large risk*
8. In your estimation, what are the chances you will be working your normal duties (at home or work) in 3 months<br>*0 1 2 3 4 5 6 7 8 9 10 (10-)*<br>*No chance Very Large Chance*
9. An increase in pain is an indication that I should stop what I'm doing until the pain decreases.<br>*0 1 2 3 4 5 6 7 8 9 10*<br>*Completely disagree Completely agree*
10. I should not do my normal work (at work or home duties) with my present pain.<br>*0 1 2 3 4 5 6 7 8 9 10*<br>*Completely disagree Completely agree*
Add all the scores to obtain the total score (For Items 3, 4, and 8 the score is 10 minus the number circled).

Supplementary figure 1. ÖMPSQ question 1. How long have you had your current pain problem? Tick (√) one. 0–1 weeks [1] 1–2 weeks [2] 3–4 weeks [3] 4–5 weeks [4] 6–8 weeks [5] 9–11 weeks [6] 3–6 months [7] 6–9 months [8] 9–12 months [9] over 1 year [10]

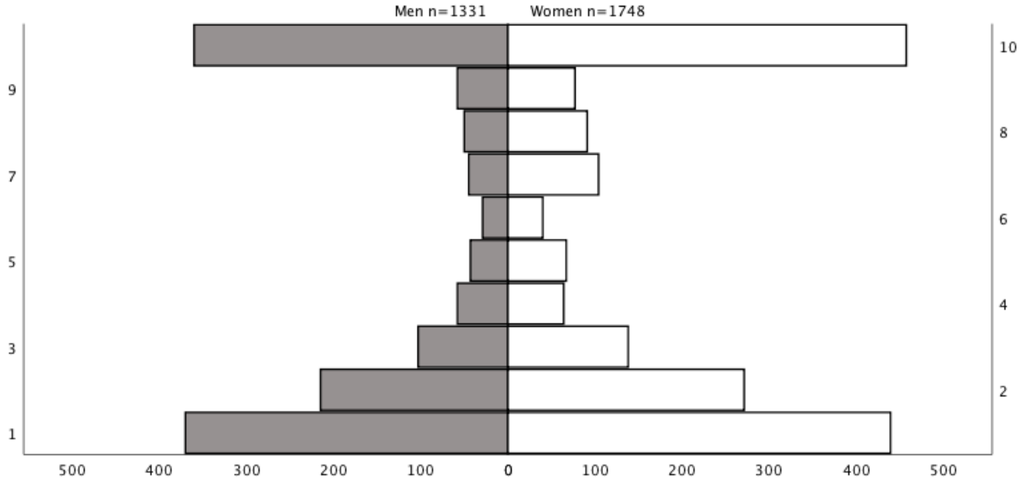

Supplementary figure 2. ÖMPSQ question 2. How would you rate the pain that you have had during the past week? Circle one. Scale 0 - 10; 0=No pain, 10= as bad as it could be.

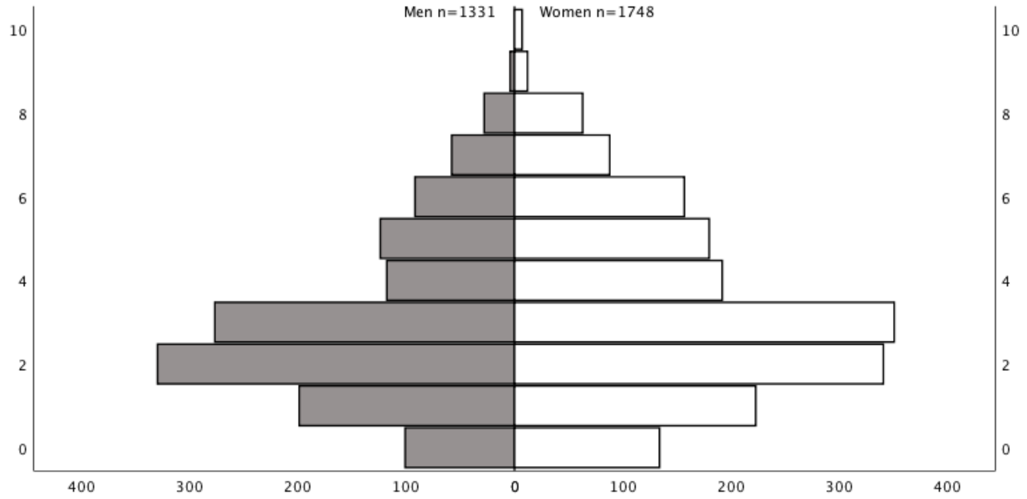

Supplementary figure 3. ÖMPSQ question 3. I can do light work (or home duties) for an hour. Scale 0-10, scores 10-x; After reversed scoring as required: 0= Without any difficulty, 10= Not at all.

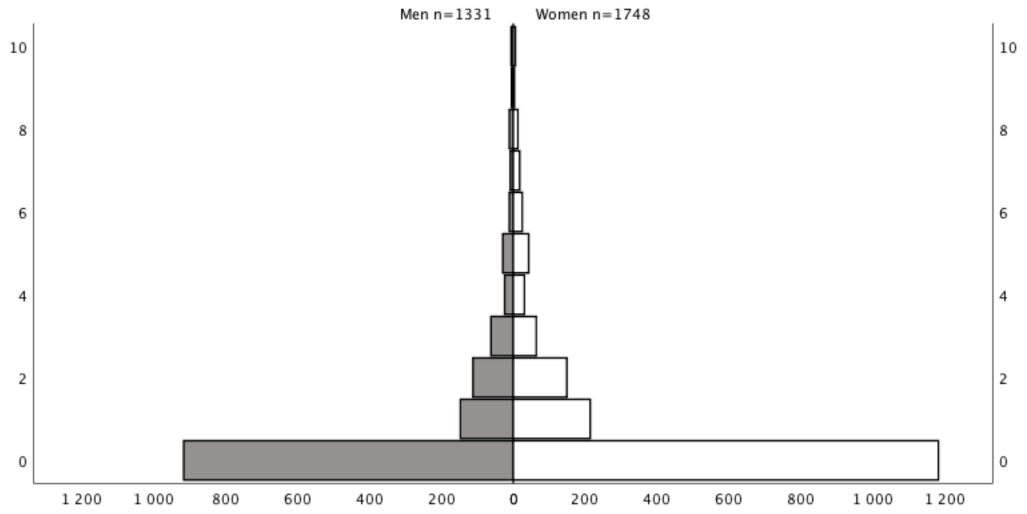

Supplementary figure 4. ÖMPSQ question 4. I can sleep at night. Scale 0-10, scores 10-x; After reversed scoring as required: 0= Without any difficulty, 10= Not at all.

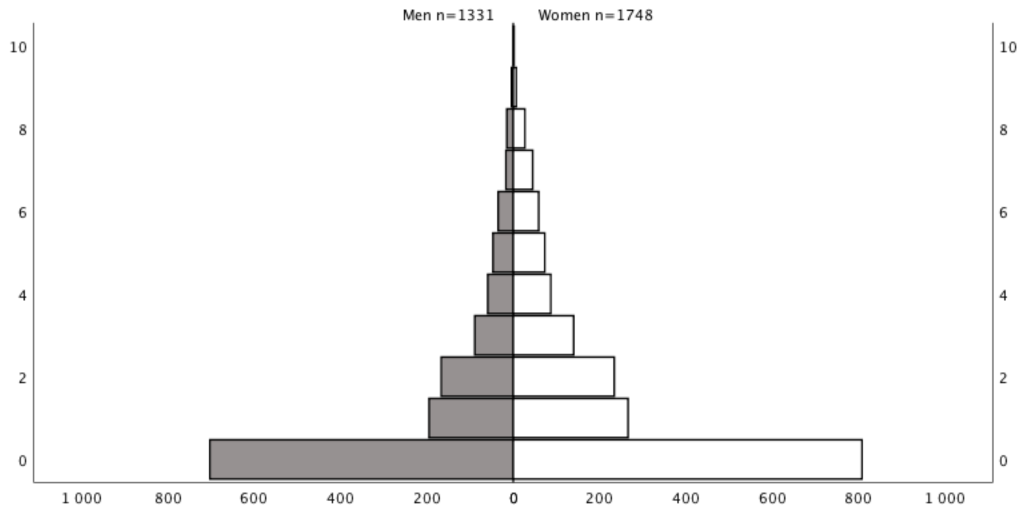

Supplementary figure 5. ÖMPSQ question 5. How tense or anxious have you felt in the past week? Scale 0-10; 0=Absolutely calm and relaxed, 10=As tense and anxious as I've ever felt.

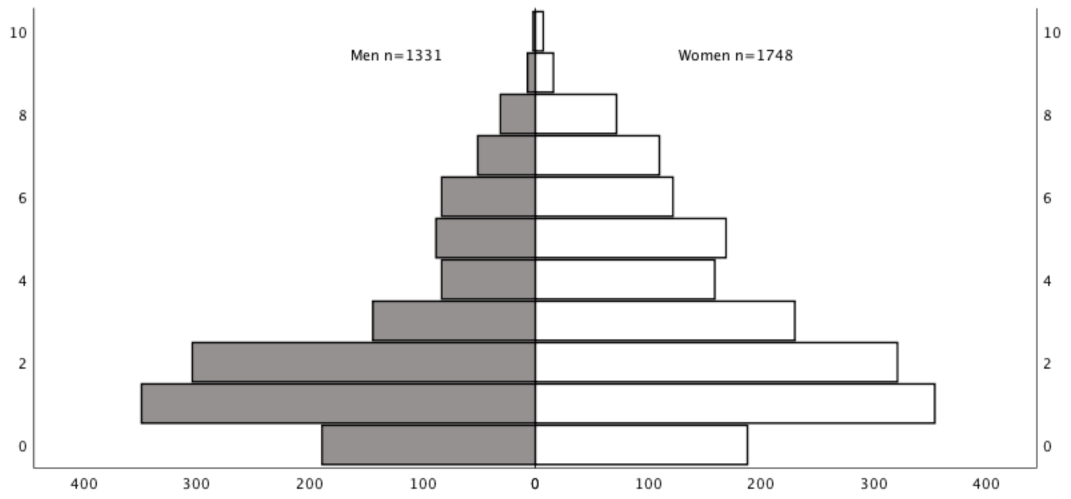

Supplementary figure 6. ÖMPSQ question 6. How much have you been bothered by feeling depressed in the past week? Scale 0-10; 0=Not at all, 10=Extremely.

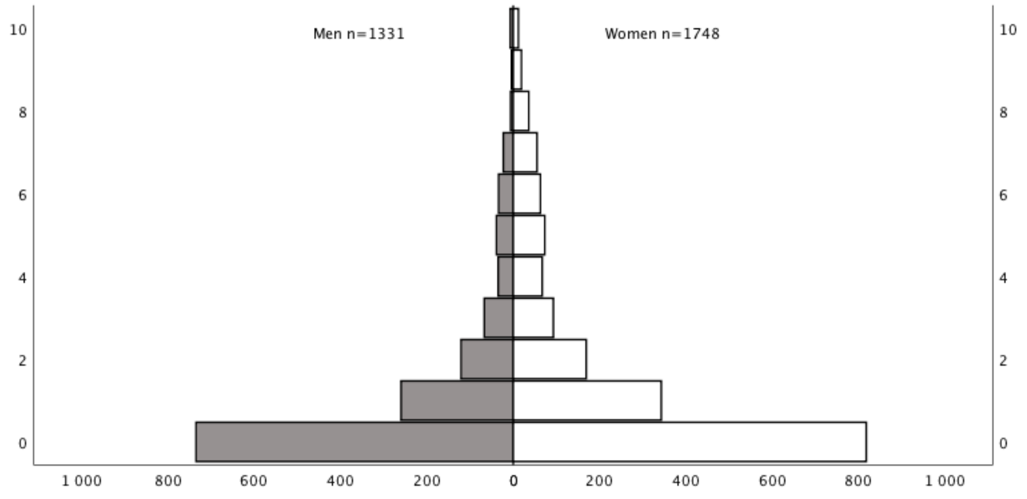

Supplementary figure 7. ÖMPSQ question 7. In your view, how large is the risk that your current pain may become persistent? Scale 0-10, 0=No risk, 10=Very large risk.

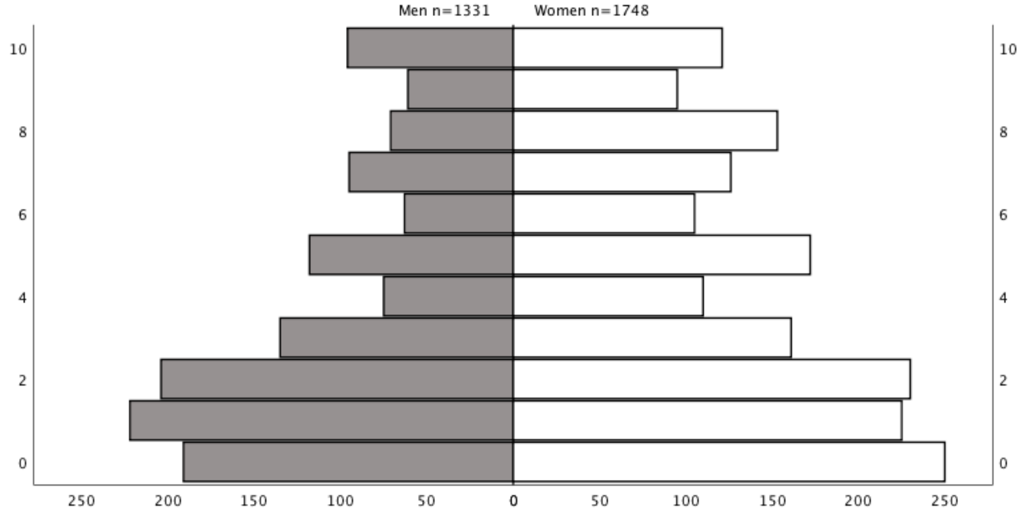

Supplementary figure 8. ÖMPSQ question 8. In your estimation, what are the chances you will be working your normal duties (at home or work) in 3 months? Scale 0-10, scores 10-x. After reversed scoring as required: 0= Very Large Chance, 10= No chance.

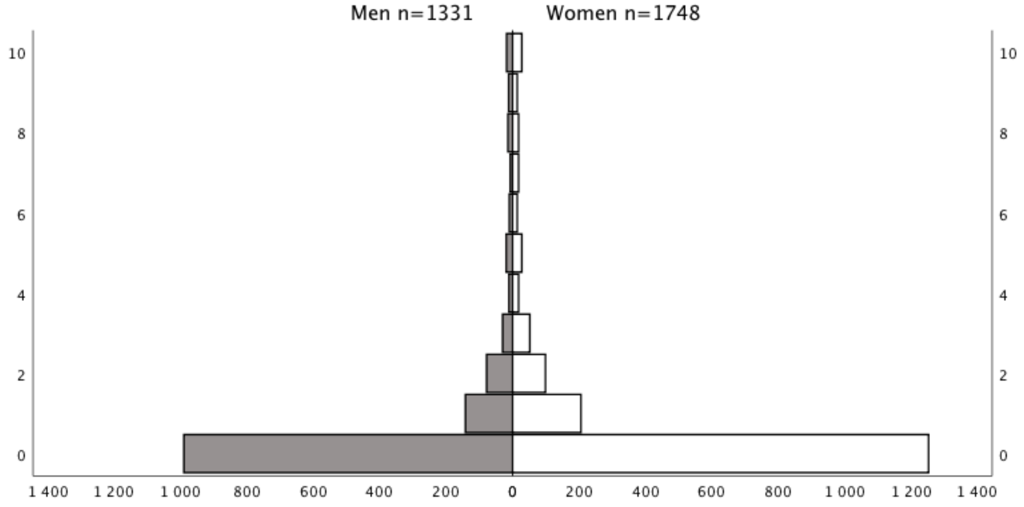

Supplementary figure 9. ÖMPSQ question 9. An increase in pain is an indication that I should stop what I'm doing until the pain decreases. Scale 0-10; 0=Completely disagree, 10=Completely agree.

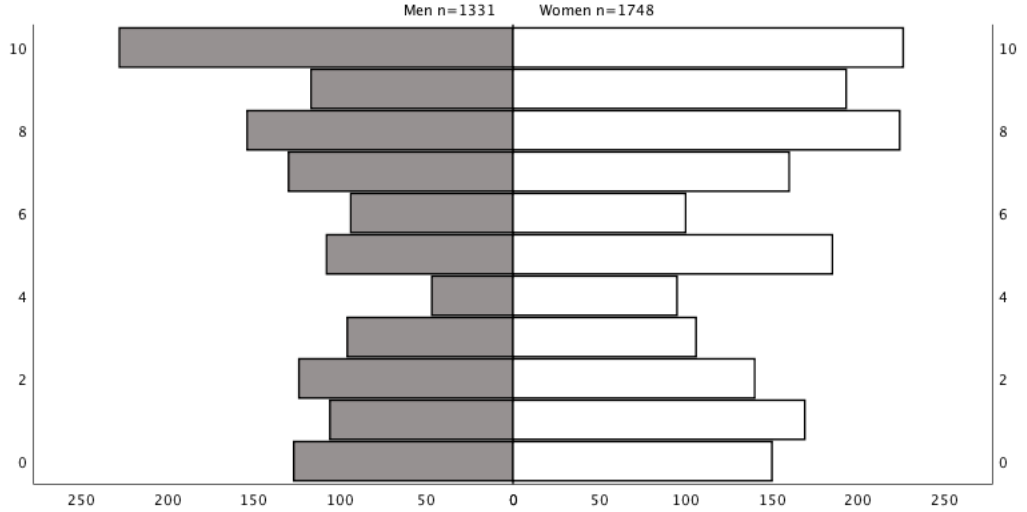

Supplementary figure 10. ÖMPSQ question 10. I should not do my normal work (at work or home duties) with my present pain. Scale 0-10; 0=Completely disagree, 10=Completely agree.

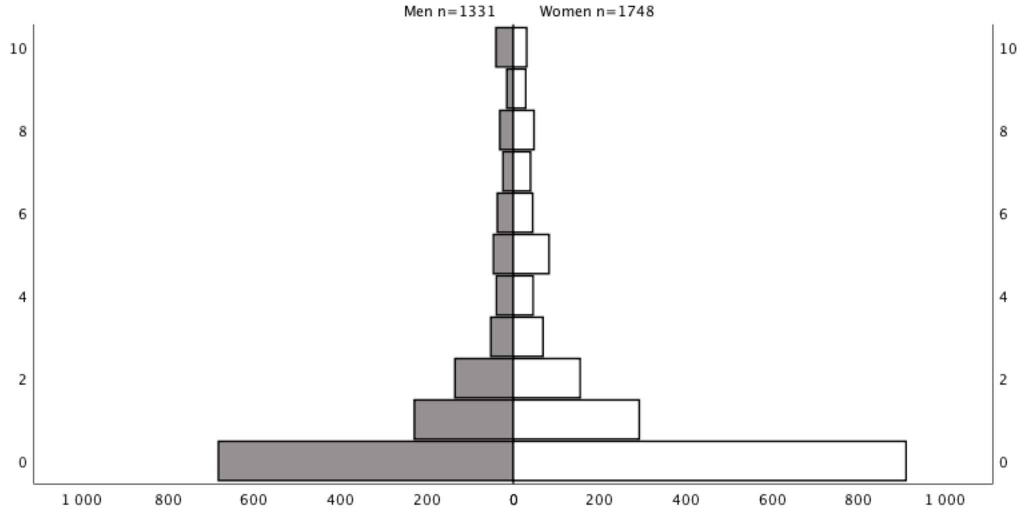

Supplement: Supplementary file 1 — Supplementary information. [file 41598_2019_57105_MOESM1_ESM.pdf]
